# Magnetic resonance imaging of knees: a novel approach to predict recombinant human growth hormone therapy response in short-stature children in late puberty

**DOI:** 10.1007/s12519-023-00758-y

**Published:** 2023-10-21

**Authors:** Xi Bai, Zhi-Bo Zhou, Xiao-Yuan Guo, Yi-Ling He, Yue-Lun Zhang, Feng-Dan Wang, Feng Feng, Hong-Bo Yang, Shi Chen, Feng-Ying Gong, Hui-Juan Zhu, Hui Pan

**Affiliations:** 1 Department of Endocrinology, Shandong Provincial Hospital Affiliated to Shandong First Medical University, Jinan, Shandong China; 2 Key Laboratory of Endocrinology of National Health Commission, Department of Endocrinology, State Key Laboratory of Complex Severe and Rare Diseases, Peking Union Medical College Hospital, Chinese Academy of Medical Science and Peking Union Medical College, Beijing, 100730 China; 3 Medical Research Center, Peking Union Medical College Hospital, Chinese Academy of Medical Sciences and Peking Union Medical College, Beijing, China; 4 Department of Radiology, Peking Union Medical College Hospital, Chinese Academy of Medical Science and Peking Union Medical College, Beijing, China

**Keywords:** Growth hormone, Growth velocity, Late puberty, Magnetic resonance imaging, Short stature

## Abstract

**Background:**

There is no appropriate tool to predict recombinant human growth hormone (rhGH) response before therapy initiation in short-stature children in late puberty. The current study aimed to explore the associations between magnetic resonance imaging (MRI) stages of the knee growth plates and rhGH response in short-stature children in late puberty.

**Methods:**

In this prospective cohort study, short-stature children in late puberty were treated with rhGH and followed up for 6 months. We proposed a novel knee MRI staging system according to the growth plate states of distal femurs or proximal tibias and divided the participants into three groups: unclosed growth plate group, marginally closed growth plate group, and nearly closed growth plate group. The primary outcomes were height gain and growth velocity (GV), which were assessed three months later.

**Results:**

Fifty participants were enrolled, including 23 boys and 27 girls. GV and height gain after 6 months of rhGH therapy decreased successively in the three groups with an increased degree of growth plate fusion, especially when grouped by proximal tibias (GV_1-3 mon_ from 9.38 to 6.08 to 4.56 cm/year, GV_4-6 mon_ from 6.75 to 4.92 to 3.25 cm/year, and height gain from 4.03 to 2.75 to 1.95 cm, all *P* < 0.001). Moreover, the MRI stages of growth plates independently served as a significant variable for GV and height gain after therapy, especially when grouped by proximal tibias (all *P* < 0.01).

**Conclusion:**

The MRI staging method is expected to be an effective tool for predicting rhGH response before therapy initiation in short-stature children in late puberty.

**Graphical abstract:**

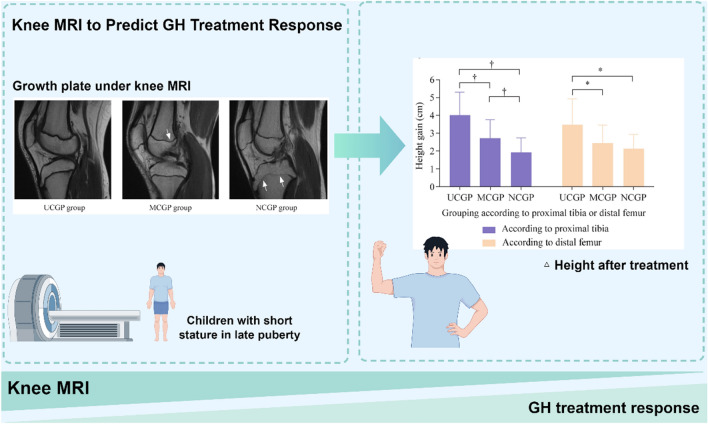

## Introduction

Recombinant human growth hormone (rhGH) therapy has been widely used in patients with short stature since its initial approval in 1985 [1–3]. However, for short-stature children in late puberty, the rhGH response is often not as good as that of prepubertal children due to their partly fused growth plates and older bone age (BA) [4–6]. In addition, the rhGH therapy response varies largely in different patients depending on age at therapy initiation, sex, etiology, and rhGH dose [2, 7–9]. Furthermore, weight gain with age means that higher rhGH doses and costs may be needed. Given all those factors, the use of rhGH in short-stature children in late puberty is a frequently discussed topic and a challenge in clinical practice. Thus, it is critical to find methods that can predict rhGH therapy response in these short-stature children in late puberty.

The growth plate, a cartilage structure located between the metaphysis and epiphysis of long bones, is one of the most important factors influencing height linear growth in children and adolescents [10]. However, as the most widely used method to evaluate bone maturity in clinical practice, X-ray-based assessment of BA cannot directly show the cartilage condition [11, 12]. Magnetic resonance imaging (MRI) is a highly accurate and nonirradiating tool for assessing bones, cartilage, ligaments, and muscles [13]. Some studies have tried to assess chronological age (CA), BA, and growth plates using different MRI sequences [12, 14–16].

Additionally, in the past 20 years, several new MRI rating scales have been proposed to evaluate knee growth plate states. Schmeling et al. and Kellinghaus et al. described a rating scale of clavicular epiphysis that compassed nonossified epiphysis (stage 1), emergence of ossification center (stage 2), partial epiphyseal–metaphyseal fusion (stage 3), total epiphyseal–metaphyseal fusion (stage 4), and disappearance of the epiphyseal scar (stage 5) [17, 18]. More specifically, stage 3 was also divided into stages 3a, 3b, and 3c, which corresponded to epiphyseal–metaphyseal fusion completion of one-third or less, one-third to two-thirds and two-thirds or more, respectively. After that, Dedouit et al. described a rating scale of the knee, in which those with a continuous horizontal cartilage line between the metaphysis and the epiphysis were divided into stage 1, 2, or 3; those with noncontinuous horizontal cartilage into stage 4, and those with total fusion into stage 5 [14]. Moreover, Vieth et al. described another rating scale of the knee, which categorized stages 2–6 according to continuous or discontinuous horizontal cartilage lines in T1-weighted and T2-weighted sequences [19]. Recently, Kvist et al. proposed a new MRI rating scale in which knee growth plate states were divided into 5 stages according to the cartilage thickness and fusion status of distal femurs and proximal tibias [12]. However, to the best of our knowledge, no study has evaluated the association between the growth plate state and rhGH therapy response.

In this study, we slightly modified and simplified the previous rating scales and proposed a novel knee MRI staging system with a combination of the previous classifications to evaluate the growth plate state. We tried to explore the association between the MRI stages of the knee growth plates and rhGH therapy response, and more importantly, to evaluate whether MRI of the knee can be a potential predictor of the rhGH response in short-stature children in late puberty.

## Methods

### Participants and study design

**Fig. 1:**
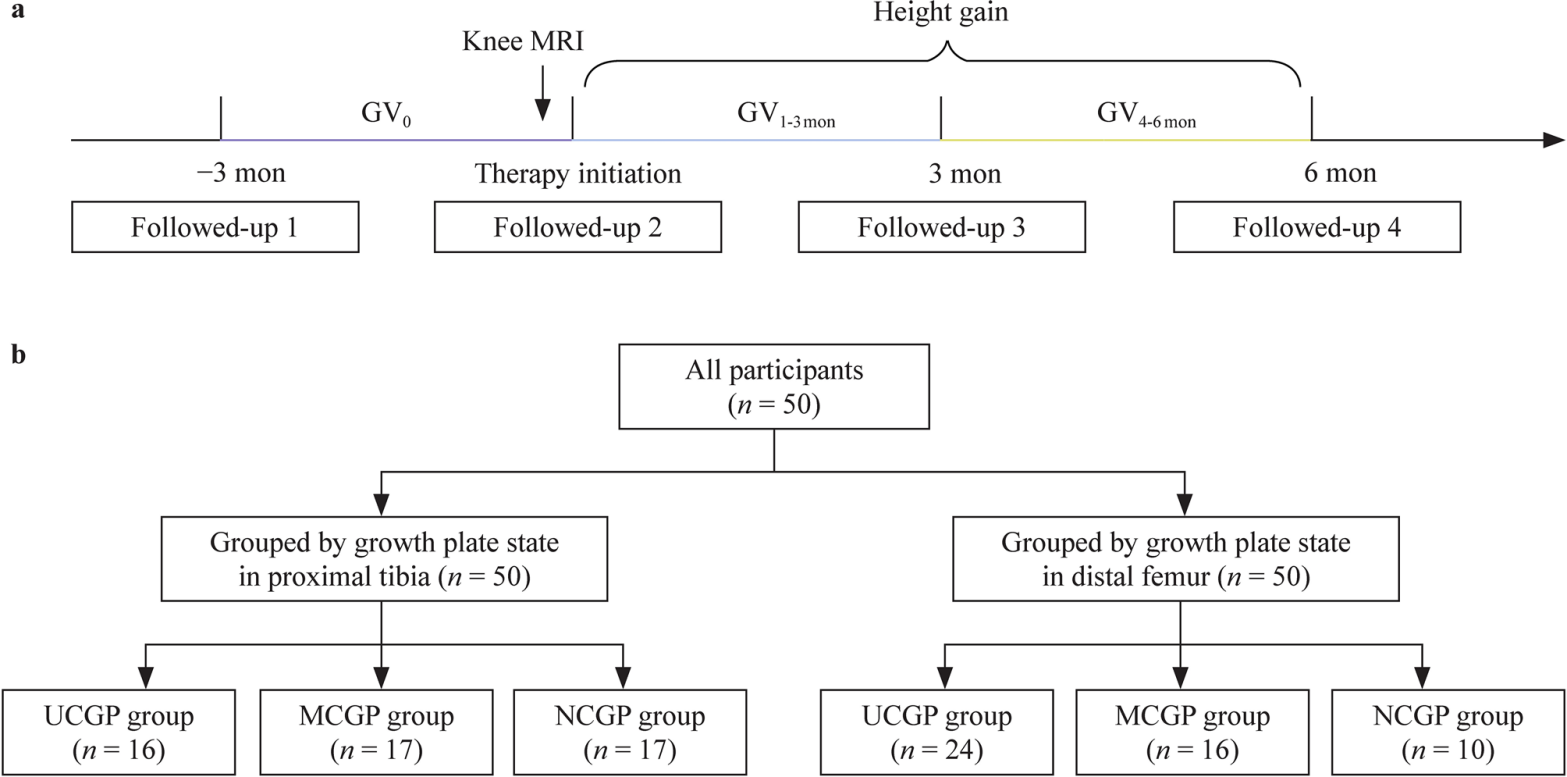
The flowchart and grouping methods of this study. **a** The flowchart of this study. In this study, four follow-up visits were performed at 3 months before therapy, therapy initiation, 3 months after therapy, and 6 months after therapy. All participants underwent a MRI of the knee on the nondominant side before therapy initiation. **b** The methods of grouping in this study. There were 50 participants divided into the UCGP, MCGP, and NCGP groups, according to the growth plate state in the proximal tibia and distal femur. *MRI* magnetic resonance imaging, *GV*_*0*_ growth velocity in 3 months before rhGH therapy, *GV*_*1-3 mon*_ growth velocity in the first-to-third months after rhGH therapy, *GV*_*4-6 mon*_ growth velocity in the fourth-to-sixth months after rhGH therapy, *UCGP* unclosed growth plate, *MCGP* marginally closed growth plate, *NCGP* nearly closed growth plate, *rhGH* recombinant human growth hormone

This study recruited 50 children with short-stature naïve to rhGH from the Department of Endocrinology in Peking Union Medical College Hospital from July 2021 to August 2022 [20]. The inclusion criteria were as follows: (1) children with short stature, which was defined as a current height < −1.5 standard deviation score (SDS), referring to the national survey data of China in 2005; or children whose predicted adult height (PAH) was lower than the target height (THt) [21]. (2) BA ≥ 14.5 years old in boys or ≥ 13.5 years old in girls according to hand–wrists referring to the Greulich and Pyle method [22]. Participants were excluded if they met any of the following exclusion criteria: (1) patients with a history of trauma or surgery on the knee of the nondominant side or those who failed to undergo knee MRI scans; (2) the knee MRI found epiphyseal cartilage in the distal femurs or proximal tibia fused completely before rhGH therapy, and (3) patients with chronic liver diseases, kidney diseases, congenital heart diseases, skeletal malformations, neurological disorders, psychiatric disorders, or chromosome abnormalities. The study design is shown in Fig. 1a. During the study, the participants were followed up every 3 months for 9 months (3 months before therapy, therapy initiation, 3 months after therapy, and 6 months after therapy). All participants underwent an MRI of the knee on the nondominant side before therapy initiation. Anthropometric measurements and laboratory examinations were evaluated at each follow-up. According to the clinical condition, the participants received rhGH therapy, a combination of rhGH and gonadotropin-releasing hormone agonist (GnRHa), or a combination of rhGH and aromatase inhibitors (AI) [23–26]. The study protocol was approved by the Ethics Committees of Peking Union Medical College Hospital on June 29, 2021 (ZS-3020). All participants and their legal guardians signed informed consent forms.

### Methods of knee magnetic resonance imaging and grouping

**Fig. 2:**
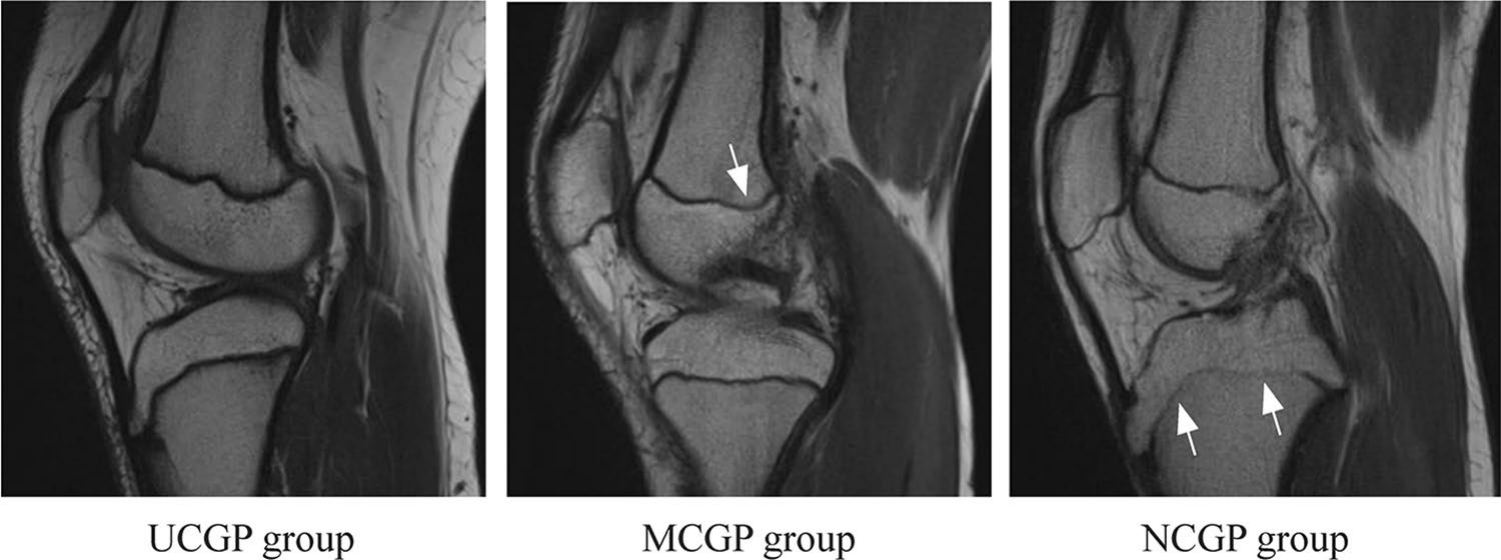
The growth plates in the UCGP, MCGP, and NCGP groups. The UCGP group is determined according to growth plate state in the proximal tibia or distal femur. If continuous cartilage signal intensity was present between the metaphysis and the epiphysis in all knee MRI images, the participant was assigned to the UCGP group (**a**). The MCGP group is determined according to growth plate state in the distal femur. If the cartilage was not continuous in any knee MRI images, but a hazy area involved was less than one-third of the growth plate between the metaphysis and the epiphysis, and the participant was assigned to the MCGP group (**b**). The NCGP group is determined according to growth plate state in the proximal tibia. If the hazy area involved was more than one-third of the growth plate between the metaphysis and the epiphysis in any knee MRI images, the participant was assigned to the NCGP group (**c**). *UCGP* unclosed growth plate, *MRI* magnetic resonance imaging, *MCGP* marginally closed growth plate, *NCGP* nearly closed growth plate

**Fig. 3:**
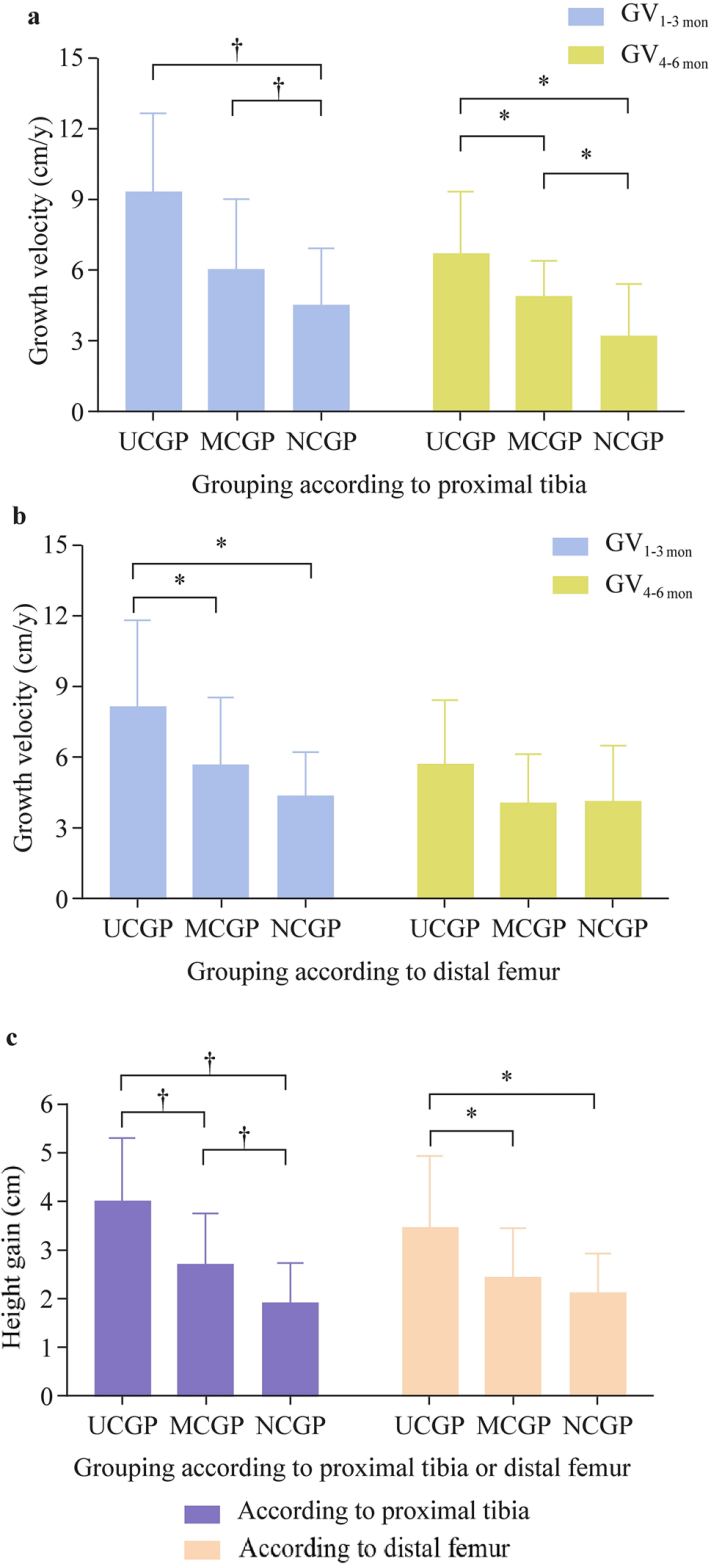
The growth velocity and height gain after 6 months of rhGH therapy. **a** Growth velocity after rhGH therapy in different groups according to the proximal tibia. GV_1-3 mon_ and GV_4-6 mon_ in the UCGP group, MCGP group, and NCGP group decreased successively (*P* < 0.001). **b** Growth velocity after rhGH therapy in different groups according to the distal femur. GV_1-3 mon_ in the UCGP group, MCGP group, and NCGP group decreased successively (*P* = 0.005). However, GV_4-6 mon_ showed no difference among the three groups (*P* = 0.074). **c** Height gain after 6 months of rhGH therapy in different groups. The height gain after 6 months of therapy sharply decreased in the UCGP group, MCGP group, and NCGP group, regardless of grouping by the proximal tibia or the distal femur (*P* < 0.001,* P* = 0.006, respectively). *GV*_*1-3 mon*_ growth velocity in the first-to-third months after rhGH therapy, *GV*_*4-6 mon*_ growth velocity in the fourth-to-sixth months after rhGH therapy, *UCGP* unclosed growth plate, *MCGP* marginally closed growth plate, *NCGP* nearly closed growth plate, *rhGH* recombinant human growth hormone, *P < 0.01, †*P* < 0.001

As described above, T1-weighted sequences were performed in the nondominant knee using 3.0T MRI before therapy to evaluate the epiphyseal cartilage in the distal femur and proximal tibia. In this study, we used a novel reference knee MRI staging system based on previous MRI staging systems with some simplifications [12, 14, 17, 18]. As shown in Fig. 1b, all participants were divided into three groups according to the growth plate state of the distal femur or proximal tibia: unclosed growth plate (UCGP) group, marginally closed growth plate (MCGP) group, and nearly closed growth plate (NCGP) group. Specifically, (1) if continuous cartilage signal intensity was present between the metaphysis and the epiphysis, the participant was assigned to the UCGP group (Fig. 2a); (2) if the cartilage was not continuous and a hazy area involved was less than one-third of the growth plate between the metaphysis and the epiphysis, the participant was assigned to the MCGP group (Fig. 2b), and (3) if the hazy area involved was more than one-third of the growth plate between the metaphysis and the epiphysis, the participant was assigned to the NCGP group (Fig. 2c). Grouping was performed according to the distal femur and proximal tibia, respectively (Fig. 1b), and the slice with the highest grade of closure of the growth plate was selected and graded. Grouping was performed independently by two investigators, and disagreements were resolved by discussion with a third investigator.

### Anthropometric measurements and laboratory examinations

Height and weight were measured in the morning by the same doctor using the same anthropometer in the Department of Endocrinology in Peking Union Medical College Hospital [20]. Height was measured three times and averaged, and differences were less than 0.2 cm. THt was calculated using THt (cm) = (height of the father + height of the mother + 13)/2 in boys and THt (cm) = (height of the father + height of the mother − 13)/2 in girls. PAH was calculated using the Bayley–Pinneau method [27, 28]. Height was transformed into height SDS based on gender and BA according to the national survey data of China in 2005 [21]. Growth velocity (GV) was calculated in this study. As shown in Fig. 1a, GV_0_ means GV in the 3 months before rhGH therapy. GV_1-3 mon_ indicates GV in the first-to-third months after rhGH therapy, and GV_4-6 mon_ indicates GV in the fourth-to-sixth months after rhGH therapy. Height gain was calculated by the height at 6 months after rhGH therapy minus the height at baseline. Weight SDS and BMI SDS were calculated according to the national survey data of China in 2005 [21].

BA was measured on the hand of the nondominant side. The radiographs for BA were independently assessed by a pediatric endocrinologist and a pediatric radiologist according to the Greulich and Pyle method [22], and disagreements were resolved by discussion with another pediatric endocrinologist.

Clinical assessments were performed by an experienced pediatric endocrinologist. Serum insulin-like growth factor-1 (IGF-1) levels, anterior pituitary function, renal and liver function, and glucose and lipid metabolism status were tested by standard protocols. Serum IGF-1 levels were transformed into IGF-1 SDS according to the reference intervals of serum IGF-1 levels in Chinese children [29].

### Statistical analysis

The demographic characteristics of the participants were compared among the three groups using one-way analysis of variance. GV_1-3 mon_, GV_4-6 mon_, and height gain were compared among the three groups using one-way analysis of variance followed by an least significant difference (LSD) post hoc test. The associations between rhGH therapy response (GV_1-3 mon_, GV_4-6 mon_, and height gain) and the MRI stages of distal femur/proximal tibia were assessed using multiple linear regressions. Model 1 only included the MRI stages. Model 2 included the MRI stages, sex, height SDS, and GV_0_. Model 3 included the MRI stages, sex, height SDS, GV_0_, rhGH dose, combination with AI, and combination with GnRHa. Statistical significance was set at *P* < 0.05. Data were analyzed using IBM SPSS software (IBM SPSS Statistics for Windows, version 26).

## Results

### Demographic characteristics

Fifty participants were enrolled in our study, including 23 boys and 27 girls. There were 16 participants in the UCGP group, 17 in the MCGP group, and 17 in the NCGP group when grouped by the growth plate state of the proximal tibia in MRI (Fig. 1b,). When grouped by distal femur, there were 24 participants in the UCGP group, 16 in the MCGP group, and 10 in the NCGP group. Fourteen participants were assigned to the different staging groups when grouped by the growth plate state of the proximal tibia and distal femur, and growth plate fusion occurred earlier in their proximal tibias than in their distal femurs, except for one participant.

The demographic characteristics of the participants are shown in Table 1. The CA and BA significantly and successively increased in the UCGP, MCGP, and NCGP groups regardless of the growth plate state of the proximal tibia or distal femur. The participants had a significantly advanced BA compared to their CA (2.20 ± 1.23 years). At the initiation of rhGH therapy, the height SDS of the entire cohort was −1.65 ± 0.66, and the BMI SDS was within the normal range. The PAH SDS (−1.41 ± 0.84) of the included participants was 0.81 ± 0.13 lower than the THt SDS (−0.60 ± 0.61). However, there were no significant differences in the baseline height SDS, THt SDS, or PAH SDS among the UCGP, MCGP, and NCGP groups, whether grouped by proximal tibia or distal femur. Additionally, no significant differences were observed in GV_0_ (3.93 ± 2.90 cm/year) among the three groups. The mean IGF-1 SDS was slightly low (−1.02 ± 0.92), with no significant differences between the groups. Moreover, all participants received rhGH therapy, of whom 14 girls were treated with a combination with GnRHa and 13 boys with a combination with AI, according to their clinical condition. The starting dose of rhGH was 0.16 IU/kg/day and was adjusted according to the GV and the serum IGF-1 level. All participants included in this study had undergone an MRI of the pituitary gland, and no abnormalities were reported.

**Table 1:** Demographic characteristics of the participants

Variables	Total	Grouped by growth plate state in proximal tibia					Grouped by growth plate state in distal femur			
		UCGP group	MCGP group	NCGP group	*P*		UCGP group	MCGP group	NCGP group	*P*
*n*	50	16	17	17	–		24	16	10	–
Male	23 (46.0%)	4 (25.0%)	10 (58.8%)	9 (52.9%)	0.121		7 (29.2%)	9 (56.3%)	7 (70.0%)	0.057
CA (y)	13.26 ± 1.55	11.86 ± 1.32	13.57 ± 1.09	14.27 ± 1.16	< 0.001		12.33 ± 1.35	14.06 ± 1.12	14.22 ± 1.34	< 0.001
BA (y)	15.46 ± 1.13	14.75 ± 1.24	15.62 ± 0.91	15.97 ± 0.91	0.004		14.98 ± 1.13	15.78 ± 0.86	16.10 ± 1.10	0.009
BA-CA (y)	2.20 ± 1.23	2.89 ± 1.39	2.04 ± 1.05	1.70 ± 0.99	0.015		2.65 ± 1.26	1.72 ± 1.06	1.88 ± 1.15	0.041
Height (cm)	155.11 ± 7.68	150.63 ± 6.67	157.34 ± 7.73	157.09 ± 7.04	0.015		152.1 ± 7.08	157.5 ± 7.12	158.5 ± 7.85	0.024
Height SDS	−1.65 ± 0.66	− 1.85 ± 0.69	−1.54 ± 0.60	−1.58 ± 0.70	0.353		−1.75 ± 0.69	−1.50 ± 0.59	−1.66 ± 0.74	0.513
THt SDS	−0.60 ± 0.61	−0.55 ± 0.47	−0.67 ± 0.67	−0.58 ± 0.68	0.842		−0.62 ± 0.56	−0.65 ± 0.56	−0.46 ± 0.80	0.722
PAH SDS	−1.41 ± 0.84	−1.71 ± 0.80	−1.16 ± 0.92	−1.38 ± 0.73	0.170		−1.56 ± 0.87	−1.19 ± 0.79	−1.41 ± 0.81	0.391
BMI SDS	0.32 ± 1.03	0.67 ± 1.03	0.01 ± 0.95	0.31 ± 1.05	0.161		0.46 ± 0.94	−0.02 ± 1.09	0.50 ± 1.08	0.287
IGF-1 SDS	−1.02 ± 0.92	−0.64 ± 1.21	−1.15 ± 0.70	−1.24 ± 0.74	0.163		−0.75 ± 1.08	−1.34 ± 0.62	−1.17 ± 0.73	0.146
GV_0_ (cm/y)	3.93 ± 2.90	3.84 ± 2.74	4.62 ± 3.02	3.34 ± 2.95	0.435		3.85 ± 2.37	4.65 ± 3.26	2.99 ± 3.43	0.365
rhGH dose (IU/kg·d)	0.16 ± 0.01	0.16 ± 0.01	0.16 ± 0.01	0.16 ± 0.01	0.757		0.16 ± 0.01	0.16 ± 0.01	0.16 ± 0.01	0.975
Combined with AI	13 (26.0%)	2 (12.5%)	4 (23.5%)	7 (41.2%)	0.190		3 (12.5%)	4 (25.0%)	6 (60.0%)	0.019
Combined with GnRHa	14 (28.0%)	8 (50.0%)	4 (23.5%)	2 (11.8%)	0.060		11 (45.8%)	2 (12.5%)	1 (10.0%)	0.026

Data represented as mean ± standard deviation or *n* (%)

*UCGP* unclosed growth plate, *MCGP* marginally closed growth plate, *NCGP* nearly closed growth plate, *CA* chronological age, *BA* bone age, *SDS* standard deviation score, *THt* target height, *PAH* predicted adult height, *BMI* body mass index, *IGF-1* insulin-like growth factor-1, *GV*_*0*_ growth velocity in three months before rhGH therapy, *rhGH* recombinant human growth hormone, *AI* aromatase inhibitor, *GnRHa* gonadotropin-releasing hormone analogue, – not available

When grouped according to BA in girls (Table 2), the BA and MRI stage of the proximal tibia were basically consistent in the smaller BA group (13.5 ≤ BA < 14.5 years, 85.7% of the MRI stages in the UCGP group). However, there was a discrepancy between the two methods in the older BA group. When the BA of girls was 15.5 years or older, 53.9% of them still had less than one-third growth plate healing. Additionally, when grouped according to BA in boys, the BA and MRI stage of the proximal tibia were consistent in the smaller BA group (14.5 ≤ BA < 15.5 years, all of the MRI stages in the UCGP and MCGP groups). Similarly, a discrepancy was observed between the two methods in the older BA group. When the BA of boys was 16.5 years or older, 46.2% of them still had less than one-third growth plate healing. Similar results were observed between the MRI stage of the distal femur and BA.

**Table 2:** The MRI stages grouped by growth plate states in the proximal tibia or distal femur per gender and bone age group

Gender	Bone age (y)	Grouped by growth plate state in proximal tibia			Grouped by growth plate state in distal femur			
		UCGP group	MCGP group	NCGP group	UCGP group	MCGP group	NCGP group	Total
Female	13.5 ≤ BA < 14.5	6 (85.7%)	0 (0.0%)	1 (14.3%)	6 (85.7%)	0 (0.0%)	1 (14.3%)	7
	14.5 ≤ BA < 15.5	3 (42.9%)	3 (42.9%)	1 (14.2%)	4 (57.1%)	2 (28.6%)	1 (14.3%)	7
	BA ≥ 15.5	3 (23.1%)	4 (30.8%)	6 (46.1%)	7 (53.8%)	5 (38.5%)	1 (7.7%)	13
Male	14.5 ≤ BA < 15.5	1 (20.0%)	4 (80.0%)	0 (0.0%)	3 (60.0%)	2 (40.0%)	0 (0.0%)	5
	15.5 ≤ BA < 16.5	1 (20.0%)	2 (40.0%)	2 (40.0%)	1 (20.0%)	3 (60.0%)	1 (20.0%)	5
	BA ≥ 16.5	2 (15.4%)	4 (30.8%)	7 (53.8%)	3 (23.1%)	4 (30.8%)	6 (46.1%)	13

Data represented as *n* (%)

*MRI* magnetic resonance imaging, *BA* bone age, *UCGP* unclosed growth plate, *MCGP* marginally closed growth plate, *NCGP* nearly closed growth plate

### Recombinant human growth hormone therapy response in the different MRI staging groups

GV_1-3 mon_ and GV_4-6 mon_ in the different MRI staging groups are shown in Fig. 3a, b. Specifically, according to the growth plate state of the proximal tibia in MRI, GV_1-3 mon_ in the UCGP group, MCGP group, and NCGP group decreased sharply from 9.38 cm/year to 6.08 cm/year and then to 4.56 cm/year (*P* < 0.001), and GV_4-6 mon_ in the three groups also decreased successively from 6.75 cm/year to 4.92 cm/year and then to 3.25 cm/year (*P* < 0.001). According to the growth plate state of the distal femur, GV_1-3 mon_ in the UCGP group, MCGP group, and NCGP group decreased successively from 8.16 cm/year to 5.70 cm/year and then to 4.39 cm/year (*P* = 0.005). However, GV_4-6 mon_ showed no difference in the three groups based on the distal femur (*P* = 0.074).

Height gain in the different MRI staging groups is shown in Fig. 3c. According to the growth plate state of the proximal tibia, the height gain after 6 months of therapy in the UCGP group, MCGP group, and NCGP group sharply decreased from 4.03 to 2.75 cm and then to 1.95 cm (*P* < 0.001). According to the growth plate state of the distal femur, the height gain in the three groups decreased successively from 3.49 to 2.46 cm and then to 2.14 cm (*P* = 0.006).

### Association between growth velocity after therapy and the MRI stages of knee

Multiple linear regressions were performed to explore the association between GV after therapy and knee MRI stage. After adjusting for sex, height SDS, GV before rhGH therapy, rhGH dose, and combination of AI or GnRHa (Model 3), the MRI stages of the proximal tibia still served as a significant variable on GV_1-3 mon_ (*β*_MCGP_ = − 3.053, *β*_NCGP_ = −3.460, *P* for trend = 0.002) and GV_4-6 mon_ (*β*_MCGP_ = − 1.879, *β*_NCGP_ = − 3.091, *P* for trend = 0.001) (Table 3). In addition, in the multivariable-adjusted model (Model 3 in Table 4), the MRI stages of the distal femur also stably served as a significant variable on GV_1-3 mon_ (*β*_MCGP_ = − 1.799, *β*_NCGP_ = − 2.578, *P* for trend = 0.034). However, GV_4-6 mon_ was not significantly associated with the MRI stages of the distal femur (*P* for trend = 0.278).

**Table 3:** The association between growth velocity after therapy and the MRI stages of the proximal tibia

Proximal tibia	GV_1-3 mom_			GV_4-6 mom_		
	Regression coefficient	*P*	*P* for trend	Regression coefficient	*P*	*P* for trend
Model 1^a^						
UCGP group	Reference	Reference	< 0.001	Reference	Reference	< 0.001
MCGP group	− 3.299 (− 5.315, − 1.283)	0.002		− 1.825 (− 3.323, − 0.327)	0.018	
NCGP group	− 4.824 (− 6.839, − 2.808)	< 0.001		− 3.496 (− 4.994, − 1.998)	< 0.001	
Model 2^b^						
UCGP group	Reference	Reference	0.002	Reference	Reference	< 0.001
MCGP group	− 3.163 (− 5.139, − 1.186)	0.002		− 1.956 (− 3.539, − 0.374)	0.017	
NCGP group	− 3.601 (− 5.743, − 1.458)	0.001		− 3.343 (− 5.059, − 1.627)	< 0.001	
Model 3^c^						
UCGP group	Reference	Reference	0.002	Reference	Reference	0.001
MCGP group	− 3.053 (− 5.068, − 1.039)	0.004		− 1.879 (− 3.504, − 0.255)	0.024	
NCGP group	− 3.460 (− 5.657, − 1.264)	0.003		− 3.091 (− 4.862, − 1.319)	0.001	

The association was assessed using multiple linear regressions. Values represented as mean (95% confidence interval)

*GV*_*1-3 mom*_ growth velocity in the first-to-third months after rhGH therapy, *GV*_*4-6 mom*_ growth velocity in the fourth-to-sixth months after rhGH therapy, *UCGP* unclosed growth plate, *MCGP* marginally closed growth plate, *NCGP* nearly closed growth plate, *MRI* magnetic resonance imaging, *SDS* standard deviation score**,** *rhGH* recombinant human growth hormone, *AI* aromatase inhibitor, *GnRHa* gonadotropin- releasing hormone analogue

^a^Model 1 only included the MRI stages

^b^Model 2 included the MRI stages, gender, height SDS, and growth velocity before rhGH therapy

^c^Model 3 included the MRI stages, gender, height SDS, growth velocity before rhGH therapy, rhGH dose, combination with AI, and combination with GnRHa

**Table 4:** The association between growth velocity after therapy and the MRI stages of the distal femur

Distal femur	GV_1-3 mom_			GV_4-6 mom_		
	Regression coefficient	*P*	*P* for trend	Regression coefficient	*P*	*P* for trend
Model 1^a^						
UCGP group	Reference	Reference	< 0.001	Reference	Reference	0.044
MCGP group	− 2.464 (− 4.512, − 0.416)	0.019		− 1.643 (− 3.234, − 0.051)	0.043	
NCGP group	− 3.774 (− 6.163, − 1.386)	0.003		− 1.607 (− 3.463, 0.249)	0.088	
Model 2^b^						
UCGP group	Reference	Reference	0.021	Reference	Reference	0.154
MCGP group	− 1.804 (−3.815, 0.208)	0.078		− 1.407 (− 3.082, 0.267)	0.097	
NCGP group	−2.760 (− 5.264, − 0.256)	0.032		− 1.257 (− 3.342, 0.828)	0.231	
Model 3^c^						
UCGP group	Reference	Reference	0.034	Reference	Reference	0.278
MCGP group	− 1.799 (− 3.844, 0.245)	0.083		− 1.122 (− 2.827, 0.583)	0.191	
NCGP group	− 2.578 (− 5.188, 0.032)	0.053		− 0.948 (− 3.125, 1.228)	0.384	

The association was assessed using multiple linear regressions. Values represented as mean (95% Confidence interval)

*GV*_*1-3 mom*_ growth velocity in the first-to-third months after rhGH therapy, *GV*_*4-6 mom*_ growth velocity in the fourth-to-sixth months after rhGH therapy, *UCGP* unclosed growth plate, *MCGP* marginally closed growth plate, *NCGP* nearly closed growth plate, *MRI* magnetic resonance imaging, *SDS* standard deviation score, *rhGH* recombinant human growth hormone, *AI* aromatase inhibitor, *GnRHa* gonadotropin-releasing hormone analogue

^a^Model 1 only included the MRI stages

^b^Model 2 included the MRI stages, gender, height SDS and growth velocity before rhGH therapy

^c^Model 3 included the MRI stages, gender, height SDS, growth velocity before rhGH therapy, rhGH dose, combination with AI and combination with GnRHa

### Association between height gain after therapy and knee magnetic resonance imaging stage

As shown in Table 5, the multiple linear regressions revealed a stable significant association between height gain after 6 months of rhGH therapy and the MRI stages of the proximal tibia in all three linear regression models (all *P* for trend < 0.001). In addition, the MRI stages of the distal femur also served as a significant variable for height gain after adjusting for sex, height SDS, GV before rhGH therapy, rhGH dose, and combination of AI or GnRHa (*P* for trend = 0.045). The height gain after therapy was significantly negatively associated with the degree of growth plate fusion in the proximal tibia and distal femur.

**Table 5:** The association between height gain and the MRI stages of the distal femur/proximal tibia

Height gain	Grouping by growth plate state in proximal tibia			Grouping by growth plate state in distal femur		
	Regression coefficient	*P*	*P* for trend	Regression coefficient	*P*	*P* for trend
Model 1^a^						
UCGP group	Reference	Reference	< 0.001	Reference	Reference	0.002
MCGP group	− 1.281 (− 2.013, − 0.549)	0.001		− 1.027 (− 1.821, − 0.232)	0.012	
NCGP group	− 2.080 (− 2.812, − 1.347)	< 0.001		− 1.345 (− 2.272, − 0.419)	0.005	
Model 2^b^						
UCGP group	Reference	Reference	< 0.001	Reference	Reference	0.023
MCGP group	− 1.271 (− 2.001, − 0.540)	0.001		− 0.797 (− 1.584, − 0.010)	0.047	
NCGP group	− 1.749 (− 2.537, − 0.961)	< 0.001		− 1.035 (− 2.006, − 0.065)	0.037	
Model 3^c^						
UCGP group	Reference	Reference	< 0.001	Reference	Reference	0.045
MCGP group	− 1.118 (− 1.872, − 0.365)	0.005		− 0.703 (− 1.516, 0.110)	0.088	
NCGP group	− 1.679 (− 2.492, − 0.866)	< 0.001		− 0.951 (− 1.951, 0.048)	0.062	

Height gain was calculated by the height after rhGH therapy for 6 months minus the height at baseline

The association was assessed using multiple linear regressions. Values represented as mean (95% confidence interval)

*UCGP* unclosed growth plate, *MCGP* marginally closed growth plate, *NCGP* nearly closed growth plate, *MRI* magnetic resonance imaging, *SDS* standard deviation score**,** *rhGH* recombinant human growth hormone, *AI* aromatase inhibitor, GnRHa gonadotropin-releasing hormone analogue

^a^Model 1 only included the MRI stages

^b^Model 2 included the MRI stages, gender, height SDS, and growth velocity before rhGH therapy

^c^Model 3 included the MRI stages, gender, height SDS, growth velocity before rhGH therapy, rhGH dose, combination with AI, and combination with GnRHa

### Adverse events

Overall, there were no significant differences in the adverse events among the three groups regardless of the growth plate state in the distal femur or proximal tibia. Twelve cases of injection site pain, hyperinsulinemia, arthralgia, and headache were reported during this study. More specifically, there were seven adverse events in the UCGP group, three in the MCGP group, and two in the NCGP group when grouped by the growth plate in the distal femur and five adverse events in the UCGP group, three in the MCGP group, and four in the NCGP group when grouped by the growth plate in the proximal tibia (*P* = 0.753 and 0.672, respectively). Of these adverse events, most were mild and transient. In addition, no serious adverse events of scoliosis, intracranial hypertension, slipped capital femoral epiphysis, or the facial features of acromegaly were reported during the study.

## Discussion

This prospective cohort study has several novel findings with important implications for short-stature children in late puberty or short predicted adult height. First, based on previous MRI staging systems, our study proposed a new MRI staging method for knee growth plate assessment, which divided the patients into three groups: UCGP group, MCGP group, and NCGP group [12, 14, 17, 18]. Our results demonstrated that GV and height gain after 6 months of rhGH therapy in the UCGP group, MCGP group, and NCGP group decreased successively, especially based on the proximal tibia growth plates (all *P* < 0.001). Moreover, after adjusting for gender, height SDS, GV before rhGH therapy, rhGH dose, and combination of AI or GnRHa, the MRI stages of growth plates still served as a significant variable on GV and height gain after therapy, especially based on the proximal tibias. These findings suggest that the MRI staging method proposed in this study is expected to be an effective tool for predicting the rhGH therapy response in short-stature children in late puberty before therapy initiation and help clinicians consider both potential height gains versus medication costs.

The use of rhGH in short-stature children in late puberty is a frequently discussed topic and a thorny issue in clinical practice. Weight gain with age means that higher rhGH doses and costs may be needed. However, early use of rhGH is an important factor in achieving height gain, resulting in large variability in the response to rhGH treatment in short-stature children in late puberty [30–35]. Given all these factors, it is particularly important to predict the response to rhGH treatment before administration in short-stature children in late puberty. As the most widely used method to evaluate bone maturity in clinical practice, X-ray-based assessment of BA was previously used to predict rhGH therapy response, but mostly in younger children under 10 years of age [36–38]. In addition, X-ray-based BA assessment cannot directly show the growth plate state, which is one of the most important factors affecting linear growth in children and adolescents [10]. Growth hormone can increase chondrogenesis in the growth plate by increasing the local production of IGF-1 to promote linear growth [1, 39].

In contrast, MRI is a highly accurate and nonirradiating approach for assessing bones, cartilage, ligaments, and muscles, which could directly show the growth plate state without the disadvantages of radiography [13]. The evaluation of the growth plate by MRI has been applied for forensic age and skeletal age estimation with good performance [40–42]. It has been suggested that receiving an additional left-hand radiological examination for BA may be unnecessary when MRI is needed for the treatment of the knee [42]. However, the association between the growth plate state and rhGH therapy response in short-stature children has not been explored before. In this study, based on previous MRI staging scales [12], we proposed a new MRI staging method for growth plate assessment according to the cartilage fusion status of the distal femur and proximal tibia. Compared with previous rating scales, our MRI staging method encompassing the UCGP, MCGP, and NCGP groups was mainly designed for prediction before rhGH treatment, which was much easier to use in clinical practice and more suitable for children in late puberty. After adjusting for multiple factors, a significant association between the MRI stages of growth plates and GV as well as height gain after therapy was still observed, suggesting that the MRI staging method of the knee growth plate might be a promising tool for predicting rhGH therapy response in short-stature children in late puberty before therapy initiation. Moreover, our results showed that the MRI stage and BA were inconsistent in some patients, especially those with older BA (boys 16.5 years or older and girls 15.5 years or older). In nearly half of these children, MRI stages still indicated less than one-third growth plate healing. The MRI staging method was of great significance in identifying these children and could bring predictive benefits to them.

Interestingly, there were 14 (28.0%) patients with inconsistent MRI stages grouped by the distal femur and proximal tibia, and all of them had a higher degree of fusion of the proximal tibia growth plate than that of the distal femur growth plate, except for one participant. Additionally, proximal tibial growth plate fusion before treatment was more strongly associated with GV and height gain after treatment in children in late puberty compared to the distal femur, suggesting that MRI stages grouped by the proximal tibia were a better predictor of rhGH therapy response. Except for the greater bone maturity of the proximal tibia found in our study, another possible explanation for the stronger association between the proximal tibia and therapy response might be the less growth remaining in the proximal tibia. A mean of 1.3 cm/year of growth from the distal femur and a mean of 0.9 cm/year from the proximal tibia occurred in short-stature children in late puberty [43]. Furthermore, the tibia is known to have stronger positive allometric growth than the femur, which might contribute to the greater impact of the proximal tibia [44].

The inclusion criterion for the participants with short stature in this study was raised to less than − 1.5 SD of the normal height, because the reference range was from the national survey data of China in 2005 [21]. Over the past 35 decades, the height increment of 19-year-old boys in China ranked first in the world, and the height increment of 19-year-old girls ranked third in the world. The average height of 19-year-old boys and girls in China also ranked first in East Asia [45]. Among them, the average height of Chinese boys reached 175.7 cm and that of Chinese girls reached 163.5 cm in 2019, which was much higher than 172.7 cm for boys and 160.6 cm for girls in 2005 [21, 45]. However, there has been a lack of data from the national survey on the height of children and adolescents in recent years, so nationwide data from 2005 are still widely used. Thus, we raised the inclusion criterion to less than − 1.5 SD of the normal height for short stature in this study.

There were several limitations of this study. First, although all the participants included had normal secretion of other pituitary hormones, with an IGF-1 SDS greater than − 2 and a normal MRI result of the pituitary gland, the participants did not undergo a definitive test for growth hormone deficiency. Given that the growth hormone secretion profile could have an impact on the dose adjustment and treatment efficacy, this issue might influence the results. Second, there was no placebo or untreated group for comparison. Randomized-controlled trials are needed to further explore the prediction of the MRI staging method on rhGH treatment benefit. In addition, raising the inclusion criterion to less than − 1.5 SD of the normal height for short stature might affect the results, leading to an overestimation of the predictive effect of the MRI stages of knees. Moreover, the difference in medication regimens contributed to the heterogeneity of the participants. Although the combination of AI or GnRHa had been adjusted for in the multiple linear regression, the bias might not be completely avoided. More participants should be included to further perform the subgroup analysis. In addition, the participants were only followed up to 6 months after rhGH treatment, with a lack of data on the final height. Future studies can explore the association between the MRI stages of knees and the final height in short-stature children in late puberty.

This study proposed a new MRI staging method for knee growth plate assessment, which is expected to be an effective tool for predicting the rhGH therapy response in short-stature children in late puberty before therapy initiation. The MRI stages of knee growth plates were strongly associated with GV and height gain after rhGH therapy, especially based on the proximal tibias. This MRI staging method can be used in clinical practice to help clinicians consider both potential height gains versus medication costs before therapy initiation.

## Data Availability

The datasets generated and/or analyzed during the current study are available from the corresponding author on reasonable request.
